# Occurrence and Toxigenic Potential of Planktonic Cyanobacterial Blooms and Benthic Mats in the Tropical Rio Grande de Comitán and Montebello Lakes National Park, Southern Mexico

**DOI:** 10.1155/ijm/6195685

**Published:** 2026-11-03

**Authors:** Aída Isabel Sánchez Salas, Angela Piedad Caro Borrero, Javier Carmona-Jiménez, Kenia Paola Márquez-Santamaria

**Affiliations:** ^1^ Postgraduate Program in Marine Science and Limnology UNAM Mexico City Mexico unam.mx; ^2^ Genetic Ecology Laboratory Department of Ecology and Natural Resources Faculty of Science UNAM Mexico City Mexico unam.mx; ^3^ River Ecosystem Laboratory Department of Ecology and Natural Resources Faculty of Science National Autonomous University of Mexico (UNAM) Mexico City Mexico unam.mx

**Keywords:** cyanobacteria, cyanoHABs, cyanoHAMs, cyanotoxins, health and ecosystem hazard, polyphasic identification

## Abstract

The tropical aquatic ecosystems of the Río Grande de Comitán and Lagunas de Montebello National Park (southern Mexico) exhibit signs of advanced eutrophication, with environmental conditions that can promote the proliferation of potentially toxic cyanobacteria. This study assessed the distribution and ecotoxicological risk of planktonic blooms and benthic mats throughout the subbasin. Using a polyphasic approach combining cultured‐based analysis, molecular detection and HPLC, we found a low‐diversity cyanobacterial community dominated by *Planktothrix agardhii*, which reached densities up to 2.2 × 10^5^ cells/mL during the warmest months. Benthic *Microcoleus* sp. and localized blooms of *Anabaenopsis elenkinii* were documented. Molecular analyses confirmed the presence of microcystin and anatoxin synthesizing genes, with high microcystin concentrations detected (67.5–3169 *μ*g/L). Seasonal analyses revealed significant differences in temperature in both lakes (H = 20.8, p < 0.001) and rivers (H = 13.1, p = 0.0098), as well as in river K_25_ (H = 10.7, p = 0.0169). PCA explained 65% and 64% of the variance in the first two axes for lakes and rivers, respectively, separating sites according to gradients in nutrients, conductivity, and other water‐quality variables. In lakes, redundancy analysis (RDA) showed that the first two axes explained 72% of the constrained variation, with total nitrogen, specific conductance, total phosphorous, catchment disturbance index, pH, and dissolved oxygen contributing to the environmental gradients structuring cyanobacterial composition. Wastewater discharges from the upper basin (Comitán) act as a continuous source of nutrients and cyanobacterial inoculant, whereas tourism and fishing activities mechanically disperse these populations across the lakes. These results demonstrate the widespread occurrence of potentially toxic cyanobacteria across the subbasin and highlight the need for early‐warning and monitoring programs, particularly in areas affected by wastewater inputs and intensive human activities.

## 1. Introduction

As common inhabitants of inland aquatic ecosystems, cyanobacteria have evolved a diverse array of adaptive strategies that enable them to tolerate and colonize a broad spectrum of environmental conditions [1, 2]. These encompass structural adaptations (e.g., diverse growth forms), reproductive adaptations (e.g., specialized cells such as akinetes, hormogonia, spores), and physiological adaptations (e.g., atmospheric nitrogen fixation, through specialized cells as heterocyst, chromatic adaptation, and secondary metabolite synthesis) [3–5]. Among these secondary metabolites, cyanotoxins are particularly prominent due to their diverse biological activities. They comprise cyclic peptides (e.g., microcystins (MC) and nodularins), alkaloids (e.g., anatoxins, cylindrospermopsins (ANA) and saxitoxins), lipopolysaccharides, and polyketides (e.g., aplysiatoxins) [6–8]. Ecologically, cyanotoxins fulfill multiple roles, such as acting as signaling molecules (e.g., MC functioning as infochemicals that regulate colony phenotypes), driving programmed cell death (apoptosis), deterring grazers, and mediating both interspecific and intraspecific competition. Furthermore, their physiological functions encompass iron regulation, maintenance of sodium homeostasis, thylakoid structural support, photosynthesis, and the regulation of nitrogen metabolism [9]. Nevertheless, the formation of toxic cyanobacterial blooms represents a severe hazard to both human health and ecosystem integrity.

Based on their specific target organs or systems in humans, cyanotoxins are generally classified into hepatotoxins (MC, nodularins, and cylindrospermopsins), neurotoxins (anatoxins and saxitoxins), cytotoxins (cylindrospermopsins), and dermatoxins (lyngbyatoxins and lipopolysaccharides) [10]. Both humans and animals (wild and domestic) face exposure to these toxins through multiple pathways [11, 12]. Primary routes include direct ingestion of contaminated water and dermal contact during recreational activities in affected water bodies, which is relevant for dermatotoxin exposure [11]. Furthermore, indirect exposure can occur through the consumption of bioaccumulated toxins in seafood (e.g., fish and mollusks) or crops irrigated with contaminated water [13].

The development of cyanobacterial blooms and the biosynthesis of cyanotoxins are influenced by complex interactions among physical, chemical, and biological factors, whose relative importance may differ between lentic and lotic ecosystems. In lakes, nutrient availability, temperature, light, and water‐column stability can shape cyanobacterial seasonal dynamics, whereas in rivers, hydrological conditions are particularly important for the establishment, persistence, and spatial distribution of benthic mats [9, 11, 14–16]. Importantly, cyanobacterial biomass does not necessarily correspond to cyanotoxin production, which may vary according to the composition of toxic and nontoxic genotypes and to environmental conditions affecting toxin biosynthesis [15–17]. In tropical and subtropical systems, persistently high temperatures and solar irradiance may influence population dynamics, whereas hydrological seasonality and water‐column stability can further affect cyanobacterial persistence and spatiotemporal distribution [18, 19]. Thus, the environmental factors associated with cyanobacterial population dynamics are not necessarily those that regulate toxin biosynthesis, as individual taxa may respond differently to combinations of biotic and abiotic conditions [20, 21].

The presence of toxic cyanobacteria in Mexico has garnered significant interest over the last two decades due to the occurrence of cyanobacterial harmful algal blooms (cyanoHABs) in tropical water bodies [22]. These systems serve as drinking water supplies and recreational areas for various urban centers in the central and southern regions of the country [23, 24]. Recently, in 2021, the maximum allowable concentrations for MC and nodularins (hepatotoxins) in drinking water were incorporated into Mexican regulations (NOM‐127‐SSA1‐2021).

Because of their immense ecological, economic, and cultural value, including the presence of Mayan settlements along riverbanks and lakeshores, the Río Grande de Comitán subbasin and the Montebello Lakes National Park are highly significant to the region. Consequently, the area was declared a protected natural reserve by presidential decree in the Diario Oficial de la Federación on December 16, 1959, and later recognized as a Wetland of International Importance (Ramsar site) in 2003 [25]. Driven by land‐use modifications and demographic expansion within the basin, there has been a notable increase in human settlements, silvopastoral activities, intensive tomato farming, and deforestation [26]. Consequently, rivers and lakes have suffered from severe water pollution exacerbated by untreated urban wastewater discharges and the presence of agricultural fertilizers and agrochemicals [27]. The lakes within the Montebello Lakes National Park have experienced an increased frequency of cyanoHABs [28–31] and have undergone a shift toward a eutrophic state since 2003 [25]. Furthermore, the occurrence of cyanobacterial harmful algal mats (cyanoHAMs) has been documented in several tributary rivers [22], suggesting that the threat posed by potentially MC‐ and anatoxin‐producing cyanobacteria is widespread across the entire basin.

The trophic state of these lakes has been linked to the presence of cyanotoxins [29]. Nevertheless, the cyanoHAB‐forming species that synthesize MC and anatoxins are also associated with the overall composition of the microbial assemblage, exhibiting greater resistance to high temperatures, ultraviolet radiation, iron and nitrogen limitation, and CO_2_ depletion [28]. Therefore, identifying the species, along with understanding their environmental preferences and potential toxicity, will be crucial for assessing the risks associated with the presence of cyanoHABs and cyanoHAMs within the subbasin. Consequently, the primary objective of this research was to characterize using the polyphasic approach the recurrent benthic and planktonic cyanobacteria across the Río Grande de Comitán subbasin and Montebello Lakes National Park, Mexico, as well as, determine their cyanotoxin‐producing potential and identify environmental patterns associated with their distribution and toxicity. The resulting data will provide valuable insights for decision‐makers and stakeholders to effectively monitor water quality and execute mitigation strategies that reduce human health risks.

## 2. Material and Methods

### 2.1. Study Area

The Río Grande de Comitán subbasin and Montebello Lakes National is located in the southeastern tropical Mexico and has an extension of 810 km^2^ (Figure 1). It lies between 16°04 ^′^–16°10 ^′^ N and 91°37–91°47 ^′^ W and spans an elevation of 1380−1740 m.a.s.l (INEGI, [32]). The climate is characterized as temperate and warm humid, with abundant summer rainfall [33]. The main tributary of the subbasin is the Río Grande, which flows through urban and agricultural areas before discharging into the Montebello Lakes National Park. The karstic origin of the region promotes groundwater circulation, connecting a complex system of numerous rivers and lakes [34]. These water bodies are interconnected by underground currents or, during the rainy season, through the flooding of adjacent areas ([35, 36]; Mota‐Covarrubias, [37]; 38]). The shallow lakes (ranging from 3 to 42 m in depth) are characterized as polymictic, whereas the deeper lakes (45–198 m in depth) are warm monomictic [25, 38]. At present, 54% of the subbasin area is devoted to agriculture and human settlements. The remaining 46% is covered by natural vegetation, comprising pine, oak, sweetgum, and cloud forests (INEGI, [32]).

**Figure 1:**
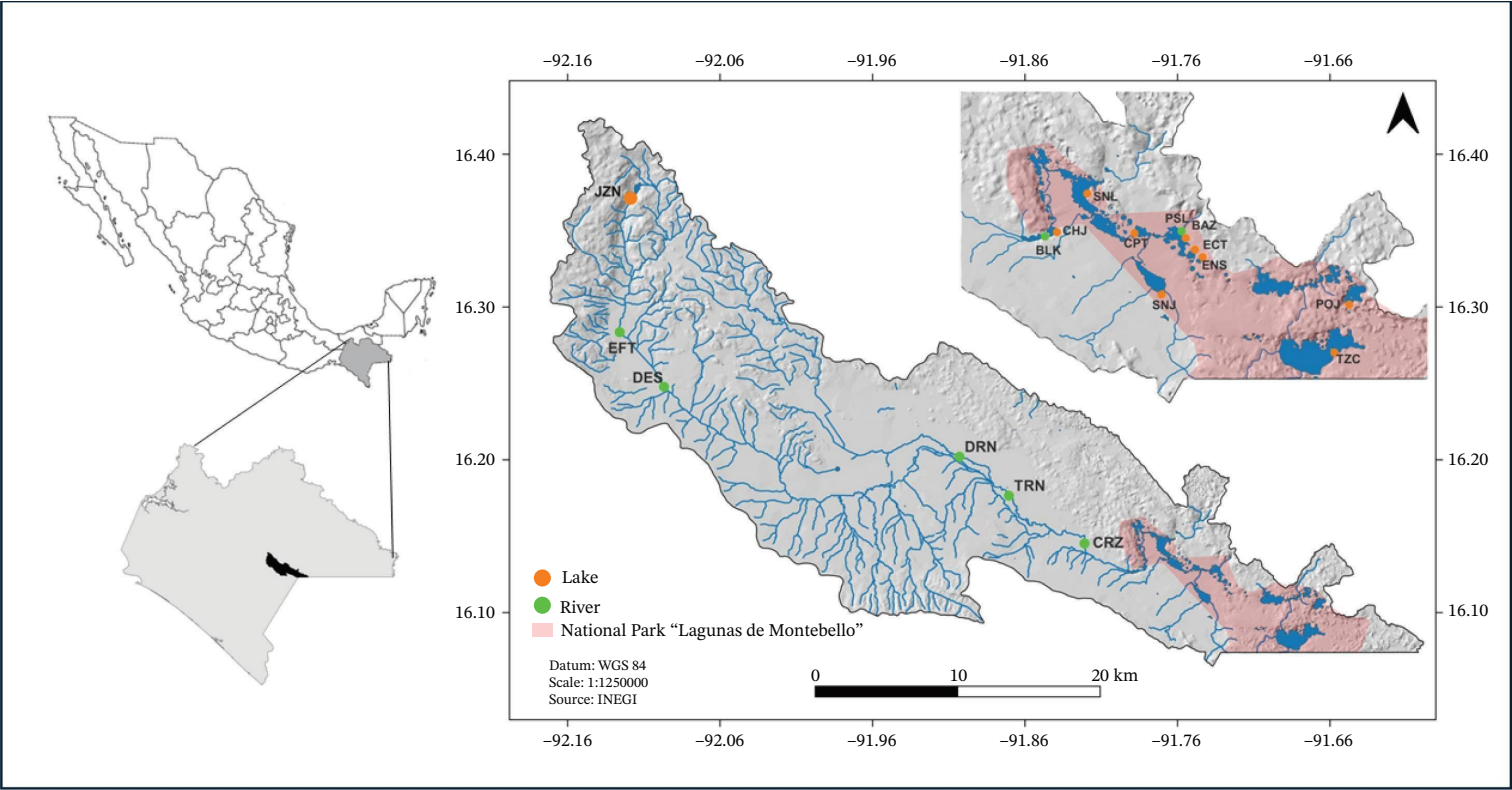
Sampling sites from the Río Grande de Comitán subbasin and Montebello Lakes National Park. High basin: Juznajab (JZN), Desamparados (DES), and Efluente (EFT). Middle basin: Drenes (DRN) and Triunfo (TRN). Low basin: Carrizal (CRZ), Balamtetik (BLK), Paso Soldado (PSL), Chaj Chaj (CHJ), San Lorenzo (SNL), Chimpotero (CPT), Bosque Azul (BAZ), Encantada (ECT) y San José (SNJ), Ensueño (ENS), Pojoj (POJ), and Tziscao (TZC).

The basin has undergone pronounced land‐use and land‐cover changes over recent decades, driven by agricultural expansion, deforestation, population growth, and urban development [39, 40]. Using satellite imagery, Rivera‐Herrera et al. [40] documented substantial changes in land use across the basin between 1974 and 2025. Throughout this period, agricultural land remained the dominant land‐use category, consistently accounting for more than 50% of the basin area. In contrast, forest cover declined by 23.5%, from 361.8 to 276.8 km^2^, whereas built‐up areas increased markedly from 5.6 to 77.5 km^2^.

#### 2.1.1. Ecological Characterization

Fieldwork was performed during the dry season (DS) (February 2022), early rainy season (ERS) (May 2022), and late rainy season (LRS) (September 2022), in 10 natural lakes and seven rivers from the Rio Grande de Comitán subbasin and Montebello Lakes National Park (Figure 1, Table 1). In situ measurements of temperature, pH, specific conductance, turbidity, and dissolved oxygen were recorded at each river and lake using a YSI 6600 multiparameter datalogger (Ohio, United States). For chemical analyses, 500 mL water samples were collected at each sampling site in sterile polypropylene bottles according to APHA [41] guidelines. All samples were kept at 4°C and analyzed in duplicate within 24 h of collection. Total nitrogen (TN) and total phosphorus (TP) concentrations were determined using a Hach DR/3900 portable spectrophotometer and a Hach DR/2000 digester (Hach, 2003).

**Table 1:** Cyanobacteria diversity and distribution in the Río Grande de Comitán subbasin and Montebello Lakes National Park. MR, morphological, ML, molecular. Acronym of the sites according to Figure 1.

Taxa	Taxonomic method	Aquatic system	Habitat	Sampling site	Isolated strain culture^a^	Sequence
Chroococcales						
*Microcystis* sp.	MR	Lake	Planktonic	SNJ, JZN		
Oscillatoriales						
*Limnothrix* sp.	MR	Lake	Planktonic	Lakes: BAZ, CHJ, CPT, ECT, SNL		
				Rivers: BLK, DRN, CRZ, TRN, PSL		
*Microcoleus* sp.	MR and ML	River	Benthonic	CRZ	CRZ I	2.1, 2.2, 62.2,
					CRZ II	4.1, 4.2, 63.8,
					CRZ III	41.11, 41.13, 41.18
*Oscillatoria* sp.	MR	Lake	Planktonic	Lakes: ECT, SNL		
				Rivers: DRN, CRZ, TRN		
*Planktothrix agardhii*	MR and ML	River and lake	Planktonic	Lakes: BAZ, CHJ, CPT, ECT, SNL	BAZ‐B	B‐22, B2‐22
				Rivers: PSL, TRN CRZ, DRE, BLK	SNL‐C	C‐22, C14‐22
					PSL‐D	D16‐22
*Raphidiopsis* sp.	MR	River	Planktonic	BLK, CRZ		
Nodosilineales						
*Persinema komarekii*	MR and ML	River	Benthonic	DRN	DRE	42.2, 42.3
Nostocales						
*Anabaenopsis elenkinii*	MR and ML	Lake	Planktonic	BAZ, CHJ, SNL	CHJ‐A	A1
*Dolichospermum* sp.	MR	Lake	Planktonic	BAZ,CHJ, CPT, ECT		

^a^Only analyzed isolated derived from cultures are marked with an ID. Blank spaces represent natural populations from the field.

#### 2.1.2. Anthropogenic Disturbance Variables at the Catchment Scale

Catchment areas for each sampling site were delineated from a digital elevation model (INEGI, [32]) using the Upslope Area tool in SAGA GIS (Version 9.3.1). Subbasin areas were then calibrated and refined by comparing them against the HydroSHEDS database [42] and the microbasin polygons defined by the local basin management plan (IMPLAN, [43]). To assess the importance of disturbances across the three different types of land use found in the basin, the Catchment Disturbance Index (CDI) was calculated [44]. In this index, urban land is assigned the highest disturbance, followed by agricultural land, and pastures. This is represented in the following heuristic, expressed mathematically: CDI = (4 × *%*of urban land) + (2 × *%*agricultural land) + (*%*pasture land). The CDI values range from 0 to 400, with higher values indicating a stronger influence of land use change on the site. To recognize the temporary physical, chemical and CDI index differences among the lakes and rivers, a principal component analysis (PCA) was performed using RStudio (4.4.3).

### 2.2. Cyanobacteria Sampling and Cultivation

Planktonic cyanobacteria were sampled across all seasons using a 4 *μ*m mesh phytoplankton net. Shoreline tows were conducted to collect 500 mL of concentrated biomass in polypropylene bottles. The objective of surface sampling was to provide information on the phytoplankton present in the accessible surface layer and is therefore suitable for subsequent monitoring and exploratory studies [45]. Morphospecies abundances were determined by analyzing quadruplicate subsamples in a Neubauer hemocytometer (0.1 mm depth, 0.0025 mm^2^ area), applying the following formula: Abundance (cells/mL) = (cell count/number of quadrants)∗10,000. Furthermore, macroscopic benthic cyanobacterial mats were gathered from discrete 10‐cm stretches within the sampled rivers. At each site and across all sampling seasons, relative abundance based on percentage coverage was determined in triplicate using a circular sampling unit of 10‐cm radius [46]. Samples of planktonic and benthic cyanobacteria were stored at 4°C prior to algal isolation and cultivation. Unialgal trichomes and colonies were isolated using a Pasteur pipette under an Olympus SC31 microscope (Japan) and inoculated onto BG11 agar plates. The resulting isolates were maintained at 20°C under a 12:12 h (light:dark) photoperiod, with a photon flux density of 40–45 *μ*mol photons m^−2^ s^−1^ provided by white, fluorescent lamps. Only six planktonic and two benthic unialgal strains were successfully isolated, with three originating from the same planktonic population. These isolates were subjected to molecular characterization (16S rRNA) and toxigenic potential assessment (via PCR and HPLC). Live cultures have been deposited and are maintained in the culture collection of the Autonomous University of Madrid, Spain.

#### 2.2.1. Taxonomic Identification

The eight cultured strains were identified using a polyphasic taxonomic approach, integrating morphological, molecular, and ecological characteristics. In contrast, the six uncultured dominant cyanobacteria were identified based on their morphological characteristics observed directly in the field‐collected material. Morphological characterization and species‐level identification were conducted relying on specialized taxonomic literature [47–49]. Microscopic analyses were carried out using an Olympus BX51 microscope equipped with an SC35 digital camera system (Japan). Molecular analyses consisted of total DNA extraction followed by the amplification of the 16S rRNA gene. Taxonomic assignments were reported to the lowest level supported by the available morphological and molecular evidence; consequently, some populations could be identified to the species level, whereas others were assigned only to the genus level.

DNA was extracted from eight cultured strains using the QIAGEN DNeasy UltraClean Microbial extraction kit. Approximately 0.15 g (wet weight) of mat material or 500 mL of planktonic samples concentrated on GF/F filters were used for DNA extraction. Genomic DNA extraction and PCR amplification of the 16S rRNA gene were performed following the protocol described by Carmona Jiménez et al. [50]. PCR amplification was carried out using primers 27F (5 ^′^‐AGAGTTTGATCCTGGCTCAG‐3 ^′^) [51] and 23Sr (5 ^′^‐CTTCGCCTCTGTGTGCCTAGGT‐3 ^′^) [52] at a final concentration of 10 pM. The resulting PCR products were purified using the Wizard SV Gel and PCR Clean‐Up System (Promega). Subsequently, cloning was performed using the pGEM‐T Easy Vector System (Promega) to ensure a broader representation of the biological diversity within the algal consortium [22, 50].

Following cloning, plasmid DNA was extracted and purified utilizing the Wizard Plus SV Minipreps DNA Purification System (Promega). The concentration of the extracted plasmids was assessed using an Epoch spectrophotometer (BioTek Instruments Inc., United States) before sequencing. Sequencing analyses were conducted at the DNA Synthesis and Sequencing Unit (USSDNA) of the Universidad Nacional Autónoma de México (UNAM) and the Genomics Unit (CAI) of the Complutense University of Madrid. The primers utilized for the sequencing reactions were T7 (5 ^′^‐TAATACGACTCACTATAGGG‐3 ^′^), SP6 (5 ^′^‐ATTTAGGTGACACTATAG‐3 ^′^) [53], and 684F (5 ^′^‐GTGTAGCGGTGAAATGCGTAGA‐3 ^′^) [54].

#### 2.2.2. Phylogenetic Analysis

Consensus sequences were assembled using the BioEdit 7.2 software (Hall, [55]) prior to analysis via the Basic Local Alignment Search Tool (BLAST, https://blast.ncbi.nlm.nih.gov/Blast.cgi). A threshold of ≥ 98% sequence identity was used to define positive matches. The integration of these molecular results with morphological identifications allowed us to taxonomically assign the analyzed cyanobacterial populations. For phylogenetic analysis, a matrix was compiled containing the newly generated sequences, similar sequences from BLAST, and additional reference sequences from related taxonomic groups (sourced from http://www.cyanodb.cz/ and https://www.ncbi.nlm.nih.gov/). Following multiple alignment in BioEdit, the dataset was manually revised in PhyDE‐1 V 0.9971 (Müller et al [56]) applying a maximum parsimony criterion. The final phylogenetic tree was estimated in MEGA V.11.0.13 (Tamura et al [57]) using the maximum likelihood method.

#### 2.2.3. Toxin Detection

To evaluate the potential toxigenicity of the dominant cyanobacteria recovered from the collected consortia, only samples from which unialgal cultures could be established were analyzed. This approach allowed the toxin biosynthesis potential to be evaluated in relation to the corresponding cyanobacterial populations while minimizing interference from other microorganisms present in the original consortia. PCR assays were carried out to screen for genes involved in the biosynthesis of major cyanotoxins, specifically the anaF, mcyE, sxtA, and cynsul genes, which are associated with anatoxin, MC, saxitoxin, and cylindrospermopsin biosynthesis, respectively. Amplification of toxin‐producing genes was carried out following the same protocol used for the 16S rRNA gene. The reaction mixture used for the PCRs included milli‐Q water, 10 × PCR buffer, Cl_2_Mg (50 mM), deoxyribonucleotide triphosphate (dNTP 50 *μ*M), (BSA, 0.1%), and DNA polymerase (Ultratools DNA Polymerase: 1 unit/*μ*L and Thermoscientific DreamTaq DNA Polymerase: 20 and 500 unit/*μ*L). Primers used to amplify anatoxins (10 pM) were atxof (5 ^′^‐TCGGAAGCGCGATCG CAAATCG‐3 ^′^) and atxar (5 ^′^‐GCTTCCTGAGAAGGT CCGCTAG‐3 ^′^) [58], whereas HEPF (5 ^′^‐TTTGGGGTTAACTTTTTTGGGCATAGTC‐3 ^′^) and HEPR (5 ^′^‐AATTCTTGAGGCTGTAAATCGGGT TT‐3 ^′^) were used to amplify MC [59]. Primers used to amplify saxitoxins were sxtAf (5 ^′^‐GCGTACATCCAAGCTGGACTCG‐3 ^′^) and sxtAr (5 ^′^‐GTAGTCCAGCTAAGGCACTTGC‐3 ^′^) [58], finally, for cylindrospermopsins we used cynsulF (5 ^′^‐ACTTCTCTCCTTTCCCTATC‐3 ^′^) and cylnamR (5 ^′^‐GAGTGAAAATGCGTAGAACTTG‐3 ^′^) (Mihali et al [60]). Milli‐Q water was included as a negative control.

#### 2.2.4. Cyanotoxin Detection and Quantification

The detection were performed via high‐performance liquid chromatography (HPLC‐UV). To assess the presence of MC and anatoxin‐a (ATX), extracts were obtained from isolated cyanobacterial cultures using 90% methanol for MC and 100% methanol for ANA. Toxin concentrations were normalized to chlorophyll‐*a* content, used as a proxy for biomass [61, 62]. The extracts were analyzed using the HPLC‐UV system (Shimazdu, model Prominence‐I, LC‐2030 LT), with a diode array detector (SPD‐M30A). An Eclipse Plus C18 column (150 × 46 mm, 5 *μ*m) (Agilent) was used as the stationary phase, whereas the mobile phases varied depending on the cyanotoxin being analyzed. For the analysis of MC‐LR, MC‐RR, and MC‐YR, an isocratic mobile phase consisting of a 32:68 (*v*/*v*) mixture of acetonitrile and aqueous acetic acid (75 mM) was utilized. For ATX analysis, a 0.2:99.8 (*v*/*v*) mixture of acetonitrile and aqueous acetic acid was employed [63]. Chromatographic separation was monitored at 239 nm for MC (MC‐LR, MC‐RR, and MC‐YR) and 228 nm for ATX, with detection limits of 0.7 and 1 ppb, respectively (Munoz et al [64]). Certified standard solutions (≥ 99% purity) of MC‐LR, MC‐RR, MC‐YR, and ATX were procured from CIFGA S.A. Laboratories. Cyanotoxin identification was based on chromatographic retention time and UV spectral characteristics in comparison with the corresponding analytical standards, and quantification (ppb) was based on peak area. These values represent the intracellular toxin content within the analyzed cyanobacterial cultures.

Because HPLC‐UV detection may be affected by matrix interference and coeluting compounds with similar UV absorbance, the results were interpreted as chemical evidence of cyanotoxin occurrence together with the molecular evidence for biosynthesis potential. This limitation is particularly relevant for MC, for which compounds with UV spectra similar to MC‐LR have been reported in cyanobacterial and environmental extracts [65].

#### 2.2.5. Statistical Analysis

Seasonal variation in physical and chemical variables was assessed separately for lake and river samples using Kruskal–Wallis tests. When significant differences among seasons were detected, pairwise Wilcoxon rank‐sum tests were performed as post hoc comparisons to identify differences between seasonal groups. *p* values were adjusted for multiple comparisons using the Benjamini–Hochberg procedure to control the false discovery rate (FDR), and differences were considered statistically significant at an FDR‐adjusted p < 0.05 [66]. PCA was used to explore temporal patterns and differences in physical, chemical, and CDI index variables within lake and river systems. Prior to PCA, environmental variables were standardized to ensure comparability among variables measured on different scales. Physical and chemical heterogeneity among sites and seasons was further explored separately for lake and river samples using hierarchical cluster analysis based on standardized environmental variables and Euclidean distances. The optimal number of clusters was determined using the average silhouette width. The relationship between cyanobacterial community composition and environmental variables was evaluated using redundancy analysis (RDA). RDA was conducted for lake samples using Hellinger‐transformed cyanobacterial abundance data and environmental variables, implemented in the *vegan* package (v2.7‐2; [67]). The overall significance of the RDA model was assessed using permutation tests, and the proportion of explained community variation was estimated using adjusted *R* ^2^. The contribution of individual environmental variables was evaluated using sequential permutation tests. Cyanobacterial taxa contributing ≥ 0.50% to the community composition were considered in the interpretation of species–environment relationships. RDA was not performed for river samples because planktonic cyanobacteria may represent transported rather than locally established populations and therefore may not reliably reflect local environmental conditions. In addition, the number of benthic cyanobacterial samples available for rivers was insufficient for multivariate analysis (n = 2).

All statistical analyses were performed in R (v4.4.3) using the *stats* and *vegan* packages through the RStudio interface. Unless otherwise specified, statistical significance was established at p < 0.05.

## 3. Results

### 3.1. Ecological Characterization

Seasonal differences were detected for temperature in both lakes (H = 20.8, FDR‐adjusted *p* < 0.001) and rivers (H = 13.1, FDR‐adjusted *p* = 0.0098), as well as for K_25_ in rivers (H = 10.7, FDR‐adjusted *p* = 0.0169) (Figure 2, Supporting Information A). In lakes, temperature differed significantly among all seasons (DS–ERS: *p* = 0.00049; DS–LRS: *p* = 0.00049; ERS–LRS: *p* = 0.0256). River temperature also differed among all seasons (DS–ERS: *p* = 0.0058; DS–LRS: *p* = 0.0313; ERS–LRS: *p* = 0.0313), whereas K_25_ differed between DS and ER (*p* = 0.020) and between DS and LRS (*p* = 0.016), but not between ERS and LRS (*p* = 0.227). No other physical or chemical variables showed significant seasonal differences after FDR correction.

**Figure 2:**
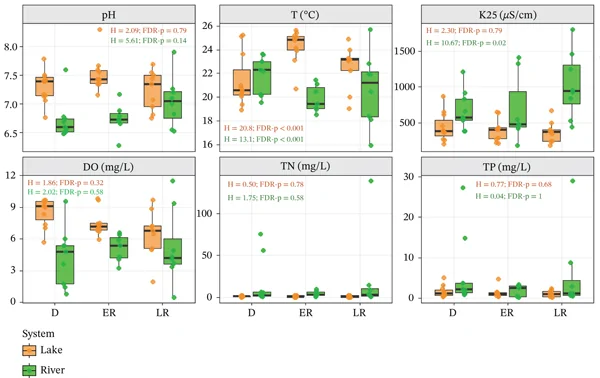
Seasonal variation in physicochemical parameters in lake and river systems. Boxplots show the distribution of pH, temperature (T), conductivity (K_25_), dissolved oxygen (DO), total nitrogen (TN), and total phosphorus (TP) across the three sampled seasons (DS, dry season; ERS, early rainy season, and LRS, late rainy season) in lakes (orange) and rivers (green). Points represent individual observations, whereas boxplots show the median and distribution of values within each season. Seasonal differences within each system were evaluated using Kruskal–Wallis tests. The *H* statistic and Benjamini–Hochberg false discovery rate (FDR)‐adjusted *p* values are shown within each panel in the corresponding system color. Significant seasonal differences after FDR correction (*p* < 0.05) were detected for temperature in both lakes and rivers and for K_25_ in rivers.

In lakes, the nutrient levels were measured as follows: TN (0.19–5.05 mg/L); TP (6.7–8.3 mg/L); and dissolved oxygen (DO, 1.93–9.83 mg/L). In rivers, the nutrient levels were measured as follows: TN (0.22–5.05 mg/L); TP (6.3–8.3 mg/L); and dissolved oxygen (DO, 0.48–11.47 mg/L). The diversity of hydromorphological characteristics and water quality in rivers and lakes was related to changes in geology and vegetation type. As well as changes in land use that range from urban and agricultural areas in the upper and middle basin of the subbasin, to the region of the protected natural area in the lower basin.

The impact of anthropogenic activities within the watershed is evidenced by elevated CDI values, ranged from 0 to 166 in lakes and from 38 to 166 in rivers. The highest CDI values were calculated in water bodies located in areas characterized by agricultural activity, tourism, and expansion of human settlements. Several of these water bodies are used for both human consumption and irrigation. The degradation observed in the upper section of the basin was consistent with the presence of *Microcystis* sp. in Lake JZN (CDI = 143). The Comitán WTP and its effluent (EFT) were also located within upper section, where proliferations of *Planktothrix agardhii* were detected.

In the middle section of the basin, extensive areas of mechanized agriculture and direct urban wastewater discharges were observed along the watercourse. The highest CDI values in this section were recorded from DRN (153) and TRN (152). Planktonic cyanobacteria detected in these rivers included *P. agardhii*, *Limnothrix* sp., *Oscillatoria* sp., and *Raphidiopsis* sp., whereas the benthic cyanobacterium *Persinema komarekii* was also detected.

In the lower section of the basin, the CRZ and BLK rivers crossed three urban centers and an area of intensive agricultural activity, with values of CDI of 136 and 166, respectively. This section also encompasses the National Park. Within these rivers and lakes, MC production was detected in *Microcoleus* sp. and *P. agardhii*, whereas anatoxin production was detected in *Anabaenopsis elenkinii*. Within the natural area, water bodies exhibited a broad of CDI values gradient. Three lakes, ENS, TZC, and POJ, showed low CDI values (0, 3, and 0, respectively) and no cyanobacteria were detected. In contrast, SNJ and JZN showed higher CDI values (74 and 143, respectively), with *Microcystis* sp. detected in both lakes.

The PCA performed for the lakes explained 65% of the total variance in the first two axes (Axis 1 = 43*%*, Axis 2 = 22*%*), revealing two main groups of sampling sites (Figure 3). The first group comprised the impacted lakes (CHJ, SNL, CPT, and ECT across all sampling dates), as well as BAZ during the DS, and was positively associated with the highest values of specific conductance (K_25_), TN, TP, and the CDI index. In contrast, the second group included the less impacted lakes (ENS, POJ, and TZC), together with samples from both rainy seasons at JZN, and was associated with the lowest values of K_25_, TN, TP, and CDI. Samples from SNJ across all seasons and BAZ during the rainy seasons were not clearly associated with either of the two main groups.

**Figure 3:**
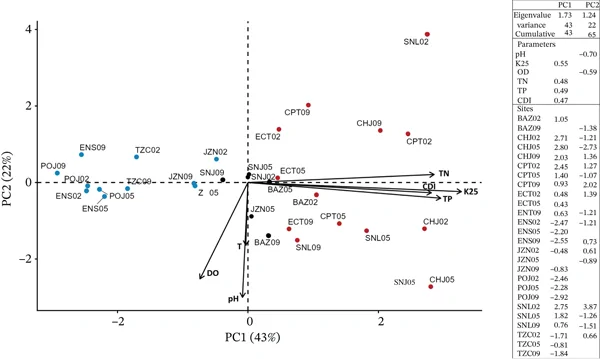
Principal component analysis (PCA) was employed to examine the physical, chemical (pH; T, temperature; DO, dissolved oxygen; K_25_, specific conductance; TN, total nitrogen; and TP, total phosphorus) parameters and the catchment area disturbance index (CDI) of the lakes within the Río Grande de Comitán subbasin and Montebello Lakes National Park. Acronym of the sites according to Figure 1. The numbers next to the names correspond to the months of the year in which the sites were sampled.

The PCA performed for the river systems explained 64% of the total variance in the first two axes (Axis 1 = 41*%*, Axis 2 = 23*%*; Figure 4), revealing three main groups of sampling sites and seasons. The first group comprised the effluent from the wastewater treatment plant (EFT), together with samples from CRZ during the DS, TRN during the dry and late seasons, and DES during the DS. These samples were associated with higher concentrations of K_25_, TN, and TP, which showed strong positive loadings on PC1. The second group included CRZ, TRN, DRN, BLK, and DES during the DS, as well as BLK during the ERS, and was primarily associated with higher pH and K_25_ values. The third group comprised BLK, CRZ, and TRN during the rainy season, together with all PSL sampling periods, and was associated with lower TN, TP, and K_25_ values. Overall, the PCA revealed an environmental gradient along PC1, ranging from samples associated with higher nutrient concentrations and conductivity to those characterized by lower TN, TP, and K_25_ values.

**Figure 4:**
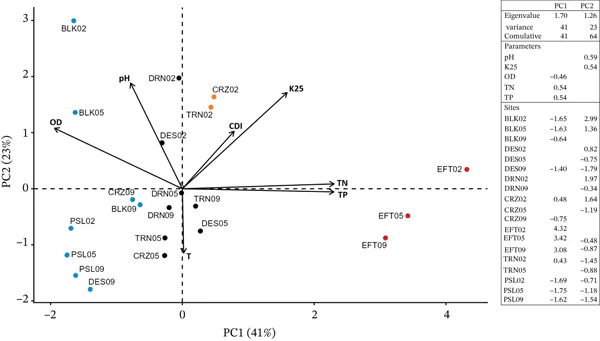
The principal component analysis (PCA) was employed to examine the physical and chemical parameters of the rivers within the Rio Grande de Comitán subbasin and Montebello Lakes National Park. The parameters included pH, temperature (T), dissolved oxygen (DO), specific conductance (K_25_), total nitrogen (TN), and total phosphorus (TP). Acronym of the sites according to Figure 1. The numbers next to the names correspond to the months of the year in which the sites were sampled.

### 3.2. Taxonomic Identification and Cyanobacteria Diversity

Of the eight cultured cyanobacteria isolates, three originated from the same sampling site (CRZI, CRZII and CRZIII), whereas the remaining five were obtained from different sites (BAZ‐B, SNL‐C, PSL‐D, DRE, and CHJ‐A). Because of the cloning processes, a total of 16 genetic sequences was obtained from these isolates, representing both planktonic and benthic cyanobacteria collected from six sites. Based on genetic affinity, as well as morphological and morphometric characteristics (Figure 5), two benthic cyanobacterial species were recognized: *Microcoleus* sp. (Figure 6), and *P. komarekii* (Figure 7) (Heidari & Hauer). Additionally, two planktonic cyanobacterial species were identified: *P. agardhii* (Gomont) Anagnostidis & Komárek and *A. elenkinii* (Miller) (Figure 8). Morphological analyses further allowed the identification of five planktonic populations related to the genera *Dolichospermum* sp., *Microcystis* sp., *Oscillatoria* sp., *Limnothrix* sp., and *Raphidiopsis* sp. (Table 1 and Figure 5). The dominant cyanobacteria populations from other collection sites that could not be successfully isolated but exhibited morphological and morphometric characteristics consistent with those of strains previously identified through the polyphasic approach were assigned to the corresponding cyanobacterial species (Supporting Information B).

**Figure 5:**
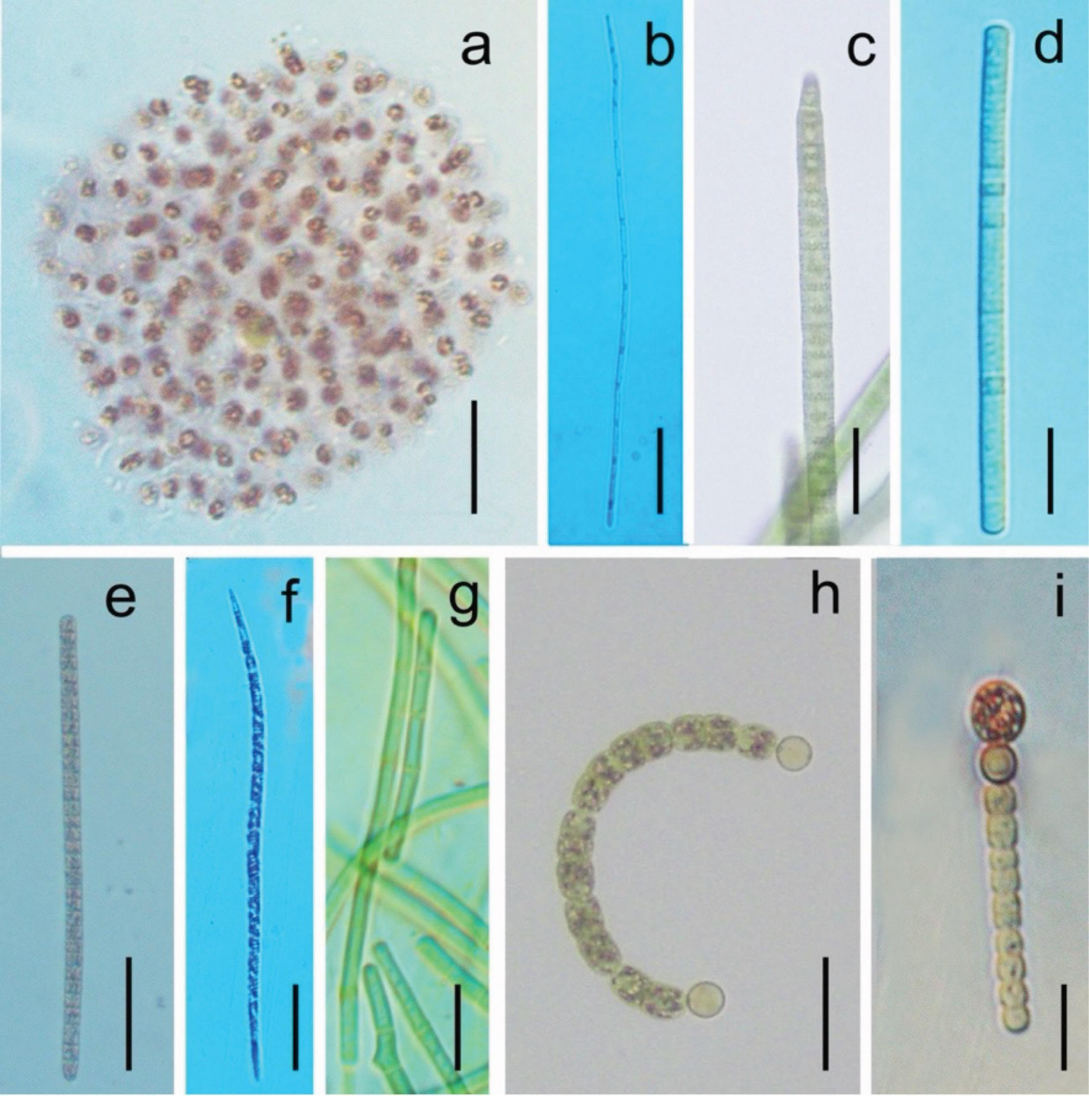
(a–j) Cyanobacteria diversity in the Río Grande de Comitán subbasin and Montebello Lakes National Park. (a) *Microcystis* sp.; (b) *Limnothrix* sp.; (c) *Microcoleus* sp.; (d) *Oscillatoria* sp.; (e) *Planktothrix agardhii;* (f) *Raphidiopsi*s sp.; (g) *Persinema komarekii;* (h) *Anabaenopsis elenkinii*; (i) *Dolichospermum* sp. Scale bar 20 *μ*m: a, b, and h. Scale bar 30 *μ*m: c, d, e, f, g, and i.

**Figure 6:**
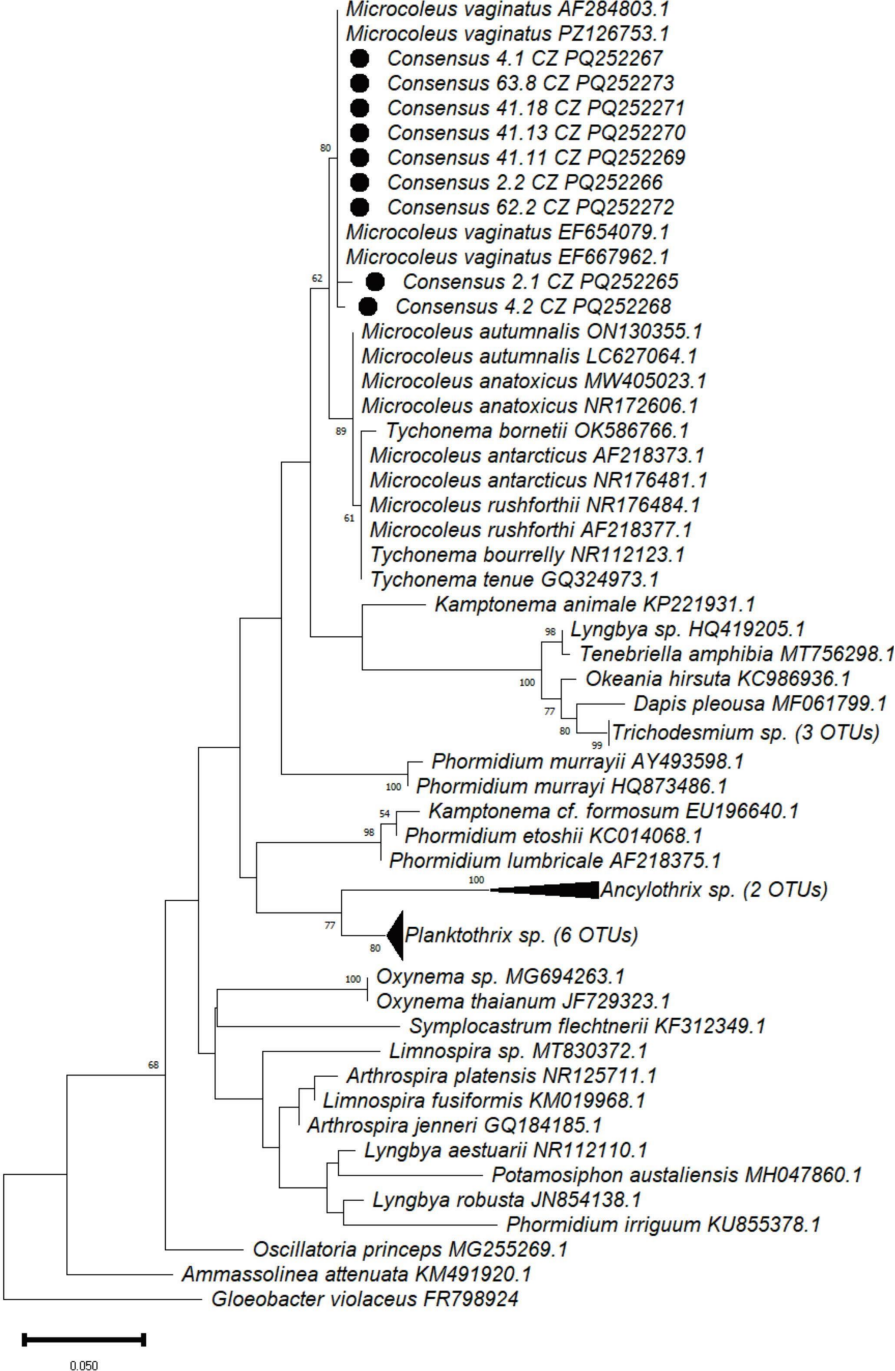
Maximum likelihood analysis for *Microcoleus* sp., based on 58 OTUs plus *Gleobacter violaceus* as the outgroup, using a 1520 nucleotide alignment of partial rRNA gene sequences. Support values bootstrap are mapped onto nodes.

**Figure 7:**
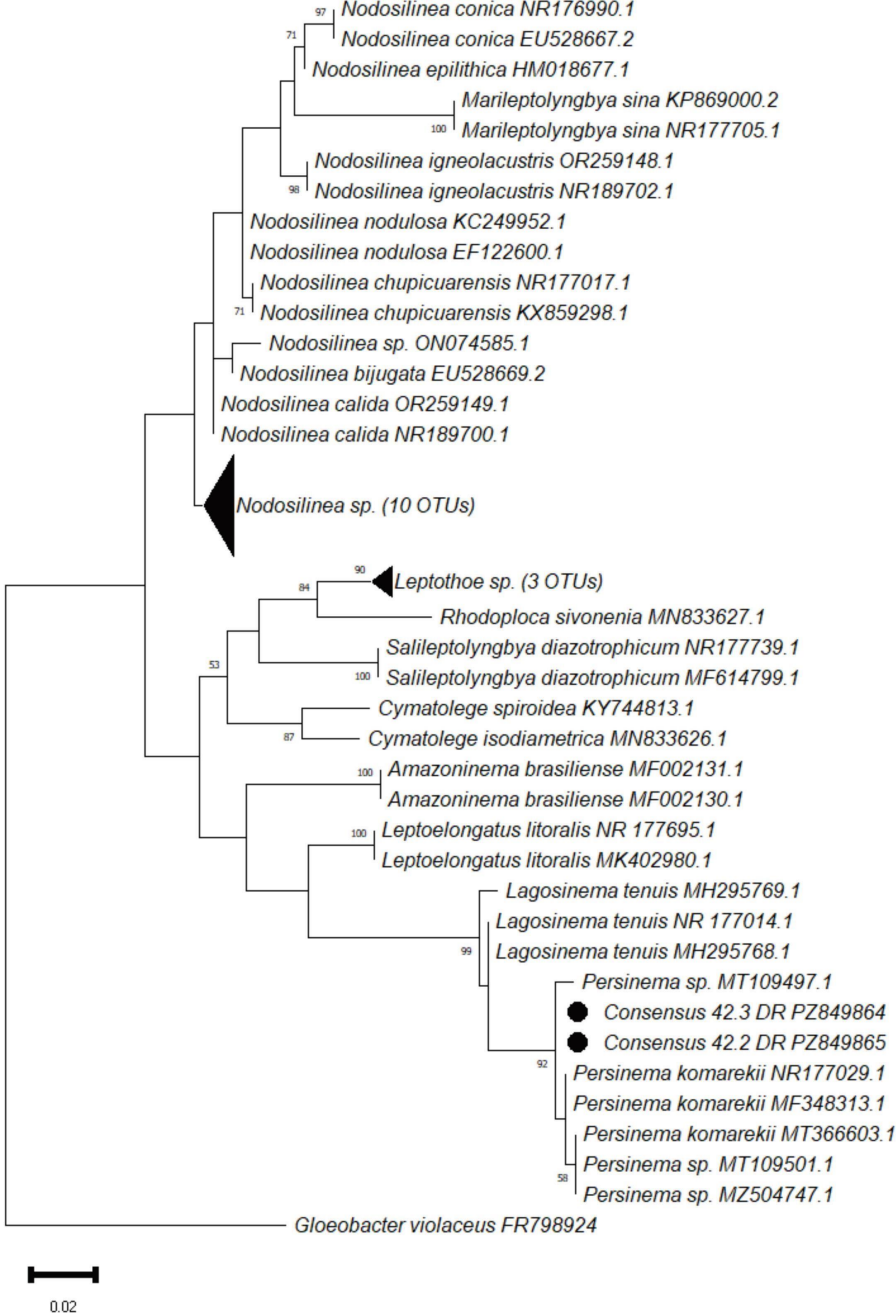
Maximum likelihood analysis for *Persinema komarekii*, based on 48 OTUs plus *Gleobacter violaceus* as the outgroup, using a 1520 nucleotide alignment of partial rRNA gene sequences. Support values bootstrap are mapped onto nodes.

**Figure 8:**
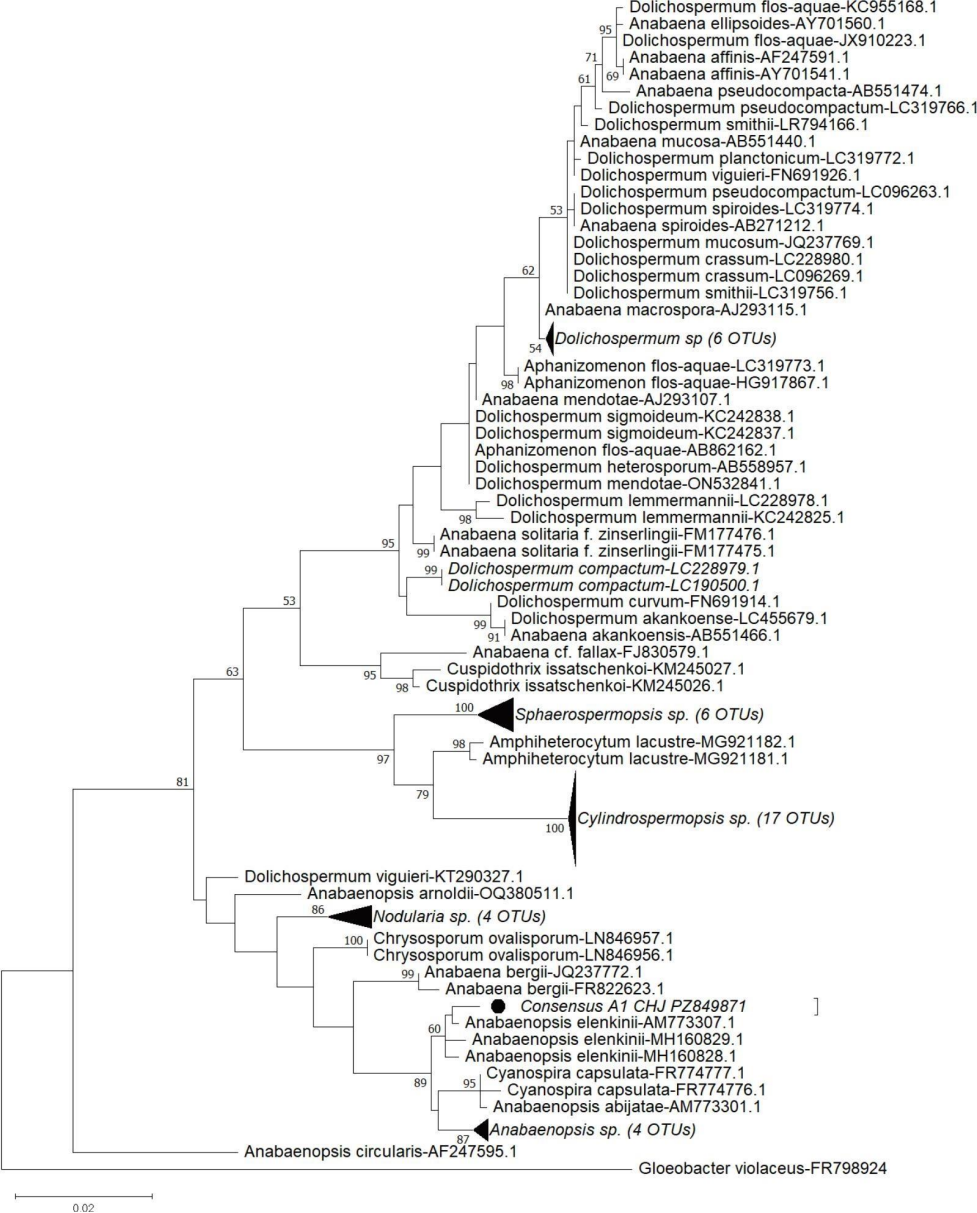
Maximum Likelihood analysis for *Anabaenopsis elenkinii*, based on 93 OTUs plus *Gleobacter violaceus* as the outgroup, using a 1520 nucleotide alignment of partial rRNA gene sequences. Support values bootstrap are mapped onto nodes.

### 3.3. Spatial and Temporal Distribution of Cyanobacteria Species

The results of the RDA between the physical and chemical parameters, sites and cyanobacteria abundance in lakes are shown in Figure 9. The first two ordination axes explained 72% of the total constrained variation. The first ordination axis (RDA1 61%) showed a negative correlation with TN (−0.86) and a positive correlation with CDI (0.57), associated with *P. agardhii* (−0.94), and two sites (SNJ, 0.71 and JZN, 0.71) with *Microcystis* sp. (1.02). Along the second ordination axis (RDA2 11%), there was a negative correlation between TP (−0.65) and CDI (−0.67) with CHJ (−1.49 to −1.68) correlated with *Limnothrix* sp. (−0.44). In contrast, *A. elenkinii*, *Dolichospermum* sp., and *Oscillatoria* sp. were located near the center of the ordination and therefore showed no clear association with a specific environmental gradient.

**Figure 9:**
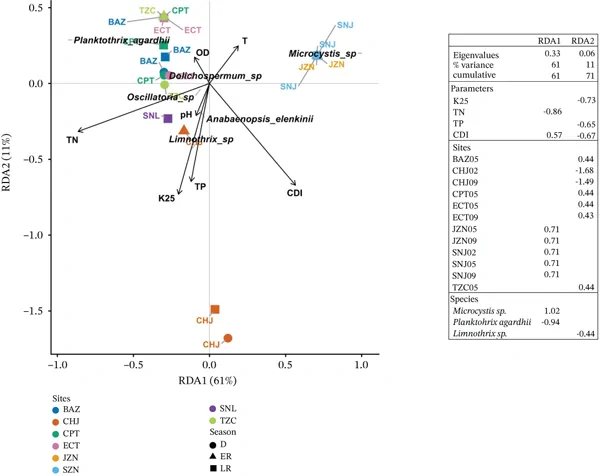
The redundancy analysis (RDA) was employed to examine the physical, chemical parameters and cyanoabcteria diversity of the lakes within the Río Grande de Comitán subbasin and Montebello Lakes National Park. The parameters included pH, temperature (T), dissolved oxygen (DO), specific conductance (K_25_), total nitrogen (TN), total phosphorus (TP) and catchment disturbance index (CDI). Acronym of the sites according to Figure 1.

### 3.4. Potential Cyanotoxins and Risk Assessment

Molecular screening via PCR confirmed the presence of the cyanotoxin producing genes *anaF* and *mcyE* in planktonic populations of *A. elenkinii* and *P. agardhii* (Table 2), with the latter also testing positive for *sxtA* in the BAZ (B) and SNL (C) sites. Chemical analysis using HPLC‐UV provided chemical evidence consistent with the occurrence of MC‐LR and MC‐RR MC congeners in all planktonic populations, showing higher concentrations of MC‐LR (Table 2). MC‐YR and ATX were not detected in either of the samples analyzed. These results were interpreted together the molecular analyses as complementary evidence of cyanotoxin occurrence and toxigenic potential. In addition, *Microcoleus* sp. cultures also exhibited toxigenic potential, as the CRZ III isolate was found to carry the *mcyE* gene.

**Table 2:** Toxicity analysis results. PCR for the anaF and anaC genes for anatoxins, mcyE (microcystins), sxtA (saxitoxins), and cyrJ (cylindrospermopsins); HPLC‐UV for anatoxins (ATX) and the LR (MC‐LR), RR (MC‐RR), and YR (MC‐YR) microcystin variants. NE, not evaluated. Acronym of the sites according to Figure 1.

Site		Cyanobacteria	PCR					HPLC			
			anaF	anaC	mcyE	sxtA	cyrJ	MC‐LR (ppb)	MC‐RR (ppb)	MC‐YR (ppb)	ATX (ppb)
Planktonic	CHJ	*Anabaenopsis elenkinii* (A)	+	—	+	—	—	550.31	67.75	—	—
	BAZ	*P. agardhii* (B)	—	—	+	+	—	3169.04	1303.55	—	—
	SNL	*P. agardhii* (C)	—	—	+	+	—	2003.99	793.10	—	—
	PSL	*P. agardhii* (D)	—	—	+	—	—	2395.91	793.10	—	—

Benthic	CRZ	*Microcoleus* sp. (CRZ I)	—	—	—	—	—	—	—	—	—
		*Microcoleus* sp. (CRZ III)	—	—	+	—	—	—	—	—	—
		*Microcoleus* sp. (CRZ IV)	—	—	—	—	—	NE	NE	NE	NE
	DRE	*Persinema komarekii*	—	—	—	—	—	NE	NE	NE	NE

Cyanobacteria were detected in 71% of the rivers and 70% of the lakes during at least one sampling campaign (Figure 10). Conversely, cyanobacteria were absent from the DES and EFT rivers, as well as lakes ENS, POJ, and TZC. *P. agardhii* was the most abundant and widely distributed species across the subbasin, spanning from the upper basin to the natural protected area and occurring in both lotic and lentic systems across 10 sites. Cultured strains of this species from three lakes yielded positive results for MC, which were confirmed through both PCR and HPLC‐UV analyses. Furthermore, a visible surface bloom was documented in lake BAZ, reaching a cell density of 2.2 × 10^5^ cells/mL (Table 3).

**Figure 10:**
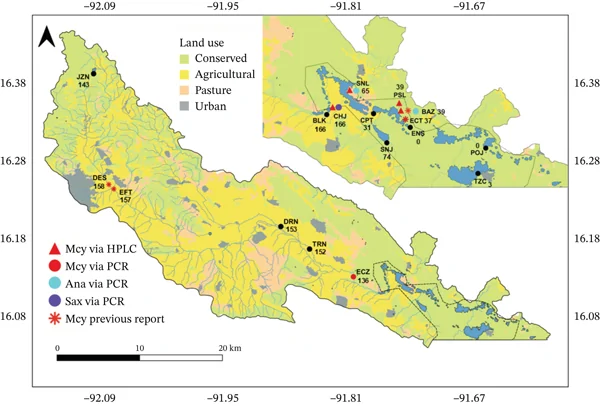
Catchment disturbance index (CDI) reported cyanotoxin occurrence, and land use at the sampling locations within the Río Grande de Comitán subbasin and Lagunas de Montebello National Park. Sites are indicated, where genes associated with microcystin (mcy), saxitoxin (sax), and anatoxin (atx) biosynthesis were detected by PCR, as well as sites where microcystins (mcy) were detected by HPLC in the present study and sites with previous reports of MC based on Fernández et al. [29] and Carmona‐Jiménez et al. [50]. The boundary of the natural protected area is indicated by a dashed line. Site acronyms correspond to those defined in Figure 1.

**Table 3:** Relative abundance of planktonic (cells/mL) and benthic (coverage %) cyanobacteria species in sampled rivers and lakes. Acronym of the sites according to Figure 1.

Planktonic																						Benthic					
Site	*Anabaenopsis elenkinii*			*Dolichospermum* sp.			*Microcystis* sp.			*Oscillatoria* sp.			*Planktothrix agardhii*			*Limnothrix* sp.			*Raphidiopsis* sp.			*Microcoleus* sp.			*Persinema komarekii*		
	Feb	May	Sep	Feb	May	Sep	Feb	May	Sep	Feb	May	Sep	Feb	May	Sep	Feb	May	Sep	Feb	May	Sep	Feb	May	Sep	Feb	May	Sep
BLK	—	—	—	—	—	—	—	—	—	—	—	—	$\begin{array}{c} 2.5\times 10^{3} \end{array}$	$\begin{array}{c} 2.3\times 10^{4} \end{array}$	$\begin{array}{c} 3.1\times 10^{3} \end{array}$	$\begin{array}{c} 3.8\times 10^{3} \end{array}$	—	—	$\begin{array}{c} 1.9\times 0^{3} \end{array}$	—	—	—	—	—	—	—	—
CRZ	—	—	—	—	—	—	—	—	—	$\begin{array}{c} 3.1\times 10^{3} \end{array}$	$\begin{array}{c} 1.3\times 10^{3} \end{array}$	—	—	$\begin{array}{c} 1.3\times 10^{3} \end{array}$	—	—	$\begin{array}{c} 6.3\times 10^{2} \end{array}$	—	—	$\begin{array}{c} 1.3\times 10^{3} \end{array}$	—	—	70	—	—	—	—
DES	—	—	—	—	—	—	—	—	—	—	—	—	—	—	—	—	—	—	—	—	—	—	—	—	—	—	—
DRN	—	—	—	—	—	—	—	—	—	$\begin{array}{c} 6.3\times 10^{3} \end{array}$	—	—	$\begin{array}{c} 1.6\times 10^{4} \end{array}$	—	—	$\begin{array}{c} 1.3\times 10^{3}\; \end{array}$	—	—	—	—	—	—	—	—	—	30	—
EFT	—	—	—	—	—	—	—	—	—	—	—	—	—	—	—	—	—	—	—	—	—	—	—	—	—	—	—
PSL	—	—	$\begin{array}{c} 1.9\times 10^{3} \end{array}$	—	—	—	—	—	—	—	—	—	$\begin{array}{c} 7.8\times 10^{4} \end{array}$	$\begin{array}{c} 5.1\times 10^{4} \end{array}$	$\begin{array}{c} 7.1\times 10^{4} \end{array}$	$\begin{array}{c} 1.1\times 10^{4} \end{array}$	—	—	—	—	—	—	—	—	—	—	—
TRN	—	—	—	—	—	—	—	—	—	$\begin{array}{c} 1.3\times 10^{4} \end{array}$	$\begin{array}{c} 6.3\times 10^{2} \end{array}$	—	$\begin{array}{c} 6.3\times 10^{2} \end{array}$	$\begin{array}{c} 6.3\times 10^{2} \end{array}$	—	$\begin{array}{c} 1.3\times 10^{3} \end{array}$	$\begin{array}{c} 1.3\times 10^{3} \end{array}$	—	—	—	—	—	—	—	—	—	—
BAZ	—	—	$\begin{array}{c} 6.3\times 10^{3} \end{array}$	—	—	$\begin{array}{c} 1.6\times 10^{4}\; \end{array}$	—	—	—	—	—	—	$\begin{array}{c} 1.4\times 10^{5} \end{array}$	$\begin{array}{c} 6.4\times 10^{4} \end{array}$	$\begin{array}{c} 2.2\times 10^{5} \end{array}$	$\begin{array}{c} 1.1\times 10^{4} \end{array}$	—	$\begin{array}{c} 2.5\times 10^{3} \end{array}$	—	—	—	—	—	—	—	—	—
CHJ	—	$\begin{array}{c} 4.5\times 10^{4} \end{array}$	—	—	—	$\begin{array}{c} 1.3\times 10^{3} \end{array}$	—	—	—	—	—	—	—	$\begin{array}{c} 3.5\times 10^{4}\; \end{array}$	$\begin{array}{c} 6.3\times 10^{2} \end{array}$	$\begin{array}{c} 1.2\times 10^{4} \end{array}$	$\begin{array}{c} 6.3\times 10^{2} \end{array}$	$\begin{array}{c} 1.9\times 10^{4} \end{array}$	—	—	—	—	—	—	—	—	—
CPT	—	—	—	—	—	$\begin{array}{c} 2.5\times 10^{3}\; \end{array}$	—	—	—	—	—	—	$\begin{array}{c} 1.1\times 10^{5} \end{array}$	$\begin{array}{c} 1.1\times 10^{5} \end{array}$	$\begin{array}{c} 1.5\times 10^{5} \end{array}$	$\begin{array}{c} 9.4\times 10^{3}\; \end{array}$	—	$\begin{array}{c} 2.5\times 10^{3} \end{array}$	—	—	—	—	—	—	—	—	—
ECT	—	—	—	—	—	$\begin{array}{c} 6.3\times 10^{2} \end{array}$	—	—	—	$\begin{array}{c} 6.3\times 10^{3} \end{array}$	—	—	$\begin{array}{c} 2.5\times 10^{4} \end{array}$	$\begin{array}{c} 6.1\times 10^{4} \end{array}$	$\begin{array}{c} 9.6\times 10^{4} \end{array}$	$\begin{array}{c} 1.3\times 10^{3} \end{array}$	—	—	—	—	—	—	—	—	—	—	—
ENS	—	—	—	—	—	—	—	—	—	—	—	—	—	—	—	—	—	—	—	—	—	—	—	—	—	—	—
JZN	—	—	—	—	—	—	—	$\begin{array}{c} 6.9\times 10^{3} \end{array}$	$\begin{array}{c} 1.1\times 10^{4} \end{array}$	—	—	—	—	—	—	—	—	—	—	—	—	—	—	—	—	—	—
POJ	—	—	—	—	—	—	—	—	—	—	—	—	—	—	—	—	—	—	—	—	—	—	—	—	—	—	—
SNJ	—	—	—	—	—	—	$\begin{array}{c} 6.3\times 10^{2} \end{array}$	8750	$\begin{array}{c} 4.4\times 10^{3}\; \end{array}$	—	—	—	—	—	—	—	—	—	—	—	—	—	—	—	—	—	—
SNL	—	—	2.5 × 10^3^	—	—	—	—	—	—	—	—	$\begin{array}{c} 2.5\times 10^{3} \end{array}$	$\begin{array}{c} 2\times 10^{4}\; \end{array}$	$\begin{array}{c} 4.5\times 10^{4}\; \end{array}$	$\begin{array}{c} 1.9\times 10^{5} \end{array}$	$\begin{array}{c} 3.8\times 10^{3} \end{array}$	$\begin{array}{c} 5.0\times 10^{3} \end{array}$	—	—	—	—	—	—	—	—	—	—
TZC	—	—	—	—	—	—	—	—	—	—	—	—	—	—	—	—	—	—	—	—	—	—	—	—	—	—	—

Overall, the occurrence of *Limnothrix* sp. and *Oscillatoria* sp. was closely associated with the lakes where *P. agardhii* was documented; however, their abundance and temporal persistence were notably lower (Table 3). The phytoplanktonic taxa *Dolichospermum* sp., *Microcystis* sp., and *A. elenkinii* exhibited a highly heterogeneous distribution and abundance across the subbasin, peaking in both frequency and abundance during the dry and warm rainy seasons. Regarding the benthic species, only *Microcoleus* sp. tested positive for MC genes, although active toxin production could not be confirmed via HPLC analysis.

## 4. Discussion

### 4.1. Cyanobacteria Diversity

The potential diversity of cyanobacteria in the subbasin amounts to 11 taxa: six identified in the present study and five previously described in the literature for this region, namely, *Limnothrix planctonica* [30], *Cyanobium* sp. [31], *Synechococcus* sp. (MC), *Cylindrospermopsis* sp. (CYN) [28], and *Planktothrix spiroides* (MC) [50]. The use of cultures combined with molecular analysis supported taxonomic identification through polyphasic approach. However, the identification of some taxa remains challenging because of the technical difficulties associated with their isolation, as well as the limited availability and reliability of reference genetic sequences for tropical cyanobacteria. This limitation is further compounded by the presence of misidentified or provisionally classified sequences in public databases, which can hinder accurate taxonomic assignment [68, 69]. Despite the relatively low cyanobacterial diversity of planktonic and benthic species across water bodies in contrast to what has been reported for other tropical lakes in the country [70–73], certain taxa were found to be frequent and widely distributed across the subbasin, in river and lake systems.

### 4.2. Ecology and Distribution

From an ecological perspective, the RGC subbasin constitutes a heterogeneous system of rivers and lakes characterized by diverse geological origins and environmental quality gradients. The thermal stability of tropical affinity (19°C–24.5°C) and the connection of the lakes with the rivers that flow through the subbasin favor the widespread and permanent distribution of cyanobacteria. As expected, the maximum proliferation of these populations was strongly associated with the warmest month of the year and a wide variation throughout the seasons in relation to TN, TP, K_25_, and CDI [18, 19]. The low correlation between physical and chemical parameters, lakes, and abundance of cyanobacteria in the RDA may be related to the broad environmental preference of the dominant taxa of the subbasin. *P. agardhii* and *Limnothrix* sp. were the most widely recorded species in both rivers (85% sites) and lakes (70% sites), occurring at 10 of the 17 sampled sites. The coexistence between both species has been recognized in recent years in the subbasin [29]. The wide distribution of *Planktothrix* is consistent with records from eutrophic tropical regions of Brazil, where it occurs under persistently high temperatures prevail year‐round, whereas variations in biovolume are mainly associated with seasonal rainfall dynamics [74, 75].

In contrast, *Microcystis* sp., *A. elenkinii, Dolichospermum* sp., and *Oscillatoria* sp. in lakes and *Microcoleus* sp., *Raphidiopsis* sp., and *P. komarekii* sp. in rivers, exhibited a distribution restricted to maximum four water bodies. The coexistence of low species diversity and extreme population densities may reflect ecosystems experiencing environmental stress, favoring the dominance of competitive taxa, as a reported in eutrophic systems [11, 74]. In the present study, *P. agardhii* was the dominant cyanobacterial taxa and accounted for much of the temporal dynamics of the subbasin. Peak abundances were observed in May and September (surpassing 2.0 × 10^5^ cells/mL in Lake BAZ), coinciding with warm conditions and potential nutrient runoff during the transition to the wet season [7]. During the warm rainy season, these lakes typically remain thermally stratified, whereas in the DS they experience water column mixing associated with seasonal turnover processes [38]. This is consistent with previous reports of *P. agardhii* dominance in regional lakes [29, 31, 50]. Where its blooms can be periodic or persistent throughout the year, occurring either as monospecific or in association with other planktonic cyanobacteria [76]. Similarly, *A. elenkinii* displayed an explosive, localized growth in Lake CHJ during May, further confirming that warm temperatures act as the primary driver for cyanobacterial blooms in this area. In this study, at the end of the rainy season, associations between *P. agardhii* and populations of *Dolichospermum* sp. (BAZ, CHJ, CPT, ECT) and *Microcystis* sp. (JZN and SNJ), were recorded in at least six lakes with oligotrophic characteristics all of which are potentially MC‐forming species. In this context, the alternation of diazotrophic Nostocales blooms, such as *Dolichospermum*, has been documented in lakes with perennial *P. agardhii* blooms [77], a phenomenon where increases in water temperature and nutrient availability determine their development [74, 75]. *Dolichospermum* sp. was detected exclusively at the end of the rainy season in four lakes (BAZ, CHJ, CPT, and ECT), suggesting marked seasonality. This pattern may be related to the life cycle of Nostocales, which can produce dormant akinetes that persist in the benthos during unfavorable conditions and germinate when environmental conditions become favorable, reintroducing vegetative cells into the water column following mixing [78]. *Microcystis* sp. was detected only in oligotrophic lakes, JZNJ and SNJE. Although this genus is more commonly associated with eutrophic systems [79], its occurrence in oligotrophic and mesotrophic ones has also been documented (Reinl et al [80]). Its low (6.3 × 10^2^ cells/mL, SNJ) or undetectable (JZN) abundance during the DS, may reflect seasonal changes in population dynamics. This pattern is consistent with the seasonal dynamics described for *Microcystis* in temperate regions, where populations typically overwinter in the benthos, resulting in very low or undetectable abundances during colder periods (Šejnohová and Maršálek, [81]).

### 4.3. Potential Cyanotoxins and Risk assessment

The occurrence of cyanobacteria with toxigenic potential in lakes and rivers of the subbasin highlights the need to considers cyanotoxin exposure as a potential risk. *P. agardhii* showed evidence for toxigenic potential, MC were chemically detected and the presence of the mcyE gene was confirmed by PCR. The agreement between molecular and chemical evidence provides support for microcystin and is consistent with previous reports under nutrient‐enriched conditions [82, 83]. In our study, the widespread occurrence of *P. agardhii* appears to be related to the higher nutrient load received by the main channel of the subbasin from its upper reaches and in both lotic and lentic systems. In the middle subbasin agricultural land use predominates (34.1%) relative to human settlements (4.9%; [26]). These conditions may favor the persistence and proliferation of cyanobacteria; although, the present study did not quantify nutrient loading or establish a causal relationship between agricultural runoff and the distribution of *P. agardhii*; previous studies have reported *P. agardhii*, *P. spiroides*, and *Planktothrix* sp. in the sedimentation ponds of the Comitán city water treatment plant [50]. Because these ponds discharge into the Río Grande de Comitán , they may represent a potential pathway for the introduction or transport of cyanobacterial biomass into the river system. The occurrence of *P. agardhii* is particularly relevant because a visible surface bloom was recorded in Lake BAZ, reaching 2.2 × 10^5^ cells/mL, the occurrence of the bloom should be considered an indicator of potential exposure rather than direct evidence of a human‐health risk caused by MC at that site, given that these waters are utilized for irrigation, fishing, and recreational purposes [84]. Previous studies in the protected area reported MC concentrations of 2.0–5.3 *μ*g/L associated with a *Planktothrix–Limnothrix–Raphidiopsis* consortium [29], indicating that MC have previously occurred in these systems. Nevertheless, direct toxin measurements during future bloom events will be necessary to determine the magnitude and temporal variability of exposure. *P. agardhii* tested positive for the sxtA gene at the BAZ and SNL sites, but the absence of detectable ATX and the lack of direct chemical confirmation of saxitoxin mean that the presence of sxtA should be interpreted as evidence of genetic potential rather than confirmed toxin production [85, 86].

The detection of the ATX gene in planktonic populations of *A. elenkinii* also indicates potential anatoxin biosynthetic capacity. However, anatoxin was not detected by HPLC‐UV. The occurrence of this species nevertheless warrants monitoring because anatoxin‐producing strains of *Anabaenopsis* have been reported growing massively in lakes [87], including tropical alkaline environments [88, 89]. *P. agardhii* (mcyE) and *A. elenkinii* (ATX) were recurrently identified in lakes located in the middle portion of the subbasin. The relationship between ecological, water physical and chemical characteristics (nutrient concentration, temperature and pH) and the occurrence of these taxa is documented across both temperate and tropical lakes, suggesting greater ecological plasticity [82, 83].

The benthic *Microcoleus* population, a known potential MC‐producing cyanobacterium [17, 90, 91], exhibited extensive proliferation, reaching a maximum coverage of 70% in the studied river system. This extensive coverage is relevant from a monitoring perspective, particularly because the population carried the mcyE gene, although active production was not chemically confirmed in this isolate. However, the occurrence or proliferation of *Microcoleus* should not be interpreted as an indicator of eutrophication by anthropogenic impact, since the literature shows evidence in various senses. For example, Wood et al. [17] showed that *Microcoleus autumnalis* dominated mats can respond to land use and nutrient enrichment, but also demonstrated that their distribution is influenced by multiple interacting environmental factors, and emphasized that toxic growths are determined by a complex interaction of multiple environmental parameters. This interpretation is further supported by recent evidence from minimally impacted, oligotrophic high‐mountain rivers, where toxic *Microcoleus* dominated mats have also been documented [23]. This ecological plasticity is partly related to the ability of *Microcoleus* mats to modify and recycle nutrients within their microenvironment, allowing their persistence under nutrient‐limited conditions [90]. Nevertheless, nutrient availability may influence the composition and distribution of particular *Microcoleus* genotypes and morphotypes, with some lineages being associated with relatively higher nitrogen and phosphorus availability [92]. Therefore, although the observed TN and TP enrichment in the studied subbasin may contribute to conditions favorable for the development of benthic cyanobacterial growths, the occurrence of abundant *Microcoleus* mats should not, by itself, be considered direct evidence of eutrophication. The framework proposed by Quiblier et al. [10] and Caro‐Borrero et al. [22] provides a useful basis for assessing benthic cyanobacterial proliferations, with coverage thresholds that can be used to define different levels of surveillance and response. According to this framework, coverage of up to 20% warrants surveillance to identify environmental conditions that may favor further proliferation, whereas coverage exceeding 20% may justify an alert level, including intensified monitoring and communication with local stakeholders. Thus, although the 70% coverage reported here warrants enhanced monitoring, its ecological significance should be evaluated together with physical, chemical, hydromorphological, and biological conditions.

The remaining cyanobacterial taxa showed heterogeneous spatial and temporal patterns. *Dolichospermum* sp., *Microcystis* sp., and *A. elenkinii* reached their highest frequency and abundance during the dry and warm‐rainy seasons, whereas *Limnothrix* sp. and *Oscillatoria* sp. were mainly associated with lakes where *P. agardhii* occurred and showed lower abundance and persistence. These patterns indicate temporal and spatial variability in cyanobacterial community composition. Environmental variables such as nutrient availability, temperature, light, and hydrological conditions can influence cyanobacterial growth and local microbial and algal community structure, potentially favoring the proliferation of toxin‐producing species and increasing the occurrence and magnitude of cyanoHABs and cyanoHAMs [22, 88, 91, 93–94]. Although the relative contribution of each factor would require targeted analyses [14, 82, 83].

In general, the MC concentrations detected by HPLC‐UV in cultures obtained in this study ranged from 67.75 to 3169 *μ*g/L. These values should be interpreted specifically as culture‐based concentrations and should not be extrapolated directly to toxin concentrations in natural waters. Under culture conditions, cyanobacterial biomass and physiological state can differ substantially from those occurring in natural populations, and toxin concentrations are influenced by environmental conditions, growth phase, and strain‐specific characteristics [14, 85]. Nevertheless, the detection of MC in cultured *P. agardhii* strains, together with the presence of mcyE, confirms that at least some populations isolated from the subbasin have the capacity to produce MC under the tested conditions.

The lower basin, which corresponds to the Natural Protected Area, tourism and artisanal fishing were among the main anthropogenic activities observed. These activities may provide potential pathways for the movement of cyanobacterial biomass between water bodies, particularly through the repeated use of boats and fishing equipment. In addition, hydrological connectivity among lakes and rivers may facilitate the passive transport of cyanobacterial cells or biomass. Nevertheless, neither hydrological transport nor boat‐ and fishing‐gear–mediated dispersal was directly evaluated in this study; therefore, these mechanisms should be considered plausible pathways rather than demonstrated drivers of cyanobacterial distribution.

Finally, the occurrence of multiple cyanobacterial taxa with reported toxigenic potential suggests that changes in ecological conditions could modify the distribution and abundance of potentially hazardous populations. The present results support the need for continued surveillance rather than demonstrating that future deterioration of the subbasin will necessarily result in increased cyanotoxin concentrations. Institutionally, these events must be promptly reported to a tripartite governance network in Mexico: (1) the National Commission of Protected Natural Areas (CONANP), as the park′s administrative authority; (2) the National Water Commission (CONAGUA), responsible for monitoring national surface water quality; and (3) the Federal Commission for the Protection against Sanitary Risk (COFEPRIS) alongside local health authorities, who possess the legal mandate to issue precautionary closures and sanitary alerts in accordance with national regulations [84].

Ultimately, local containment and monitoring measures within the lakes may be insufficient if upstream sources of nutrient enrichment are not addressed. Our findings highlight the need for improved wastewater treatment and management in the upper subbasin, particularly for discharges associated with Comitán city, to reduce nutrient inputs and potentially limit conditions that favor the proliferation and downstream transport of cyanobacterial populations.

The planktonic and benthic dynamics within the subbasin reveal an ecosystem undergoing severe environmental stress and advanced eutrophication. This condition has facilitated the ecological monopoly of highly competitive taxa with broad ecological plasticity, notably the recurrent blooms of *P. agardhii* and benthic growths of *Microcoleus* sp., which are strongly catalyzed by rising temperatures and nutrient runoff.

The use of complementary methods for cyanotoxin detection proved to be a critical strategy, allowing for the validation of cyanobacteria harboring genes for anatoxin and/or MC synthesis, even when these toxins were not detected via HPLC. Although our data do not provide evidence to determine the mechanisms underlying this discrepancy, toxin production can vary depending on environmental and physiological conditions and their interactions. These finding highlights the importance of combining molecular and chemical approaches for comprehensive monitoring cyanotoxin. The molecular confirmation of MC and synthesizing genes in dominant taxa, coupled with notably high toxin concentrations detected in vitro (up to 3169 *μ*g/L), validates the system′s toxigenic potential. If such concentrations were reached during natural surface blooms, they would represent a critical sanitary hazard for irrigation, artisanal fishing, and recreational activities.

The cyanoHABs and cyanoHAMs issue is strongly driven by human activities at the watershed level. In the upper basin, discharges from the Comitán city water treatment plant act as a continuous source of nutrients and *Planktothrix* spp. Simultaneously, tourism and fishing activities in the lower basin mechanically disperse cyanobacterial populations among the natural lakes. There is an urgent need to shift from passive monitoring to an early warning system within the Lagunas de Montebello National Park. An integrated management approach combining upstream pollution control, continuous cyanobacterial and cyanotoxin monitoring, and timely communication with local stakeholders would provide a more effective strategy for protecting the ecological integrity and water uses of this ecosystem. This requires the coordinated effort of federal authorities (CONANP, CONAGUA, and COFEPRIS) to manage preventive closures during high‐risk events.

## 5. Conclusions

The planktonic and benthic dynamics within the subbasin indicate a system characterized by substantial environmental heterogeneity and nutrient enrichment, with recurrent cyanobacterial proliferations. This condition has facilitated the ecological monopoly of highly competitive taxa with broad ecological plasticity, notably the recurrent blooms of *P. agardhii* and benthic growths of *Microcoleus* sp. Seasonal analyses showed significant differences in temperature in both lakes and rivers, as well as in K_25_ among river seasons, indicating temporal variation in environmental conditions. Multivariate analyses further revealed distinct environmental gradients associated with nutrient concentrations, conductivity, pH, dissolved oxygen, and CDI. In lakes, RDA indicated that these environmental gradients explained a substantial proportion of the constrained variation in cyanobacterial community composition.

The use of complementary methods for cyanotoxin detection proved critical characterizing the toxigenic potential of the cyanobacterial community. Molecular analysis detected genes associated with anatoxin and/or MC synthesis in several taxa, even when these toxins were not detected via HPLC. This discrepancy highlights the importance of combining molecular and chemical approaches, as the presence of toxin biosynthesis genes does not necessarily indicate active toxin production under all environmental or physiological conditions.

The detection of microcystin and anatoxin synthesizing genes in dominant taxa, coupled with notably high toxin concentrations detected in vitro (up to 3169 *μ*g/L), indicates a substantial toxigenic potential within the system. However, these concentrations should not be directly extrapolated to natural surface waters. Continued monitoring of both cyanobacterial abundance and cyanotoxin concentrations is therefore necessary to assess potential risks associated with recreational activities, fishing, irrigation, and other local water uses.

The spatial patterns observed in the PCA were consistent with differences in environmental conditions among sites and the prevalence of cyanoHABs and cyanoHAMs, with wastewater‐impacted locations associated with higher nutrient and conductivity values (upper basin), whereas less impacted sites were associated with lower values. These patterns, together with the distribution of cyanobacterial taxa, highlight the importance of watershed‐scale processes in shaping cyanobacterial communities. However, the present analyses identify associations rather than demonstrating direct causal relationships between wastewater inputs, human activities, and cyanobacterial proliferation.

Given the recurrent occurrence of potentially toxic cyanobacteria and the detection of cyanotoxins and toxin‐related genes, an integrated monitoring and early‐warning strategy is warranted for the Lagunas de Montebello National Park. An integrated management approach combining upstream pollution control, continuous cyanobacterial and cyanotoxin monitoring, and timely communication with local stakeholders would provide a more effective strategy for protecting the ecological integrity and water uses of this ecosystem. Coordination among relevant environmental and public health authorities would support evidence‐based management responses during periods of elevated cyanobacterial abundance or toxin concentrations.

## Author Contributions

A.I.S.S.: conceptualization, methodology, formal analysis, investigation, writing—original draft, writing. A.P.C.B.: conceptualization, methodology, formal analysis, investigation, writing—original draft, writing—review and editing, project administration, funding acquisition. J.C‐J.: conceptualization, methodology, formal analysis, supervision, investigation, writing—review and editing, project administration. K.P.M‐S.: methodology; formal analysis, writing—review and editing.

## Funding

This study was supported by the CYTED Ciencia y Tecnología para el Desarrollo [10.13039/501100008441; TALGEN‐TOX (920PTE0102)]; Fundación Gonzalo Río Arronte (10.13039/501100020728; A‐408); Dirección General de Asuntos del Personal Académico, Universidad Nacional Autónoma de México [10.13039/501100006087; PAPIIT‐DGAPA‐UNAM (IA20125)].

## Conflicts of Interest

The authors declare no conflicts of interest.

## Supporting information


**Supporting Information** Additional supporting information can be found online in the Supporting Information section. File S1: Supporting description: For further detailed information on the physicochemical parameters evaluated in rivers and lakes during each month of sampling, please consult Supporting Information A. Likewise, all information regarding the morphometric characteristics of the dominant cyanobacteria identified in this study—which complements the photographic composition in Figure 5 of the text—can be found in Supporting Information B.

## Data Availability

The data that supports the findings of this study are available in the Supporting Information of this article.
