# Supplementary material for: Occurrence and Toxigenic Potential of Planktonic Cyanobacterial Blooms and Benthic Mats in the Tropical Rio Grande de Comitán and Montebello Lakes National Park, Southern Mexico
Source: Int J Microbiol. 2026 Nov 3;2026:6195685. doi: 10.1155/ijm/6195685 (PMC13731977; doi:10.1155/ijm/6195685)
Supplement: Supplementary file 1 [file IJM-2026-6195685-s001.docx]

**Supplementary material A**. Physical and chemical parameters evaluated in rivers and lakes. *T*, temperature; *TDS*, total dissolved solids; *K25*, specific conductance; *DO*, dissolved oxygen; *TN,* Total Nitrogen; *TP* Total Phosphorous; CDI, Catchment Disturbance Index.

| **System** | **Site** | **pH** | | | **T (°C)** | | | **K_25_ (μS/cm)** | | | **DO (mg/L)** | | | **TDS (ppm)** | | | **TN (mg/L)** | | | **TP (mg/L)** | | | **CDI** |
| --- | --- | --- | --- | --- | --- | --- | --- | --- | --- | --- | --- | --- | --- | --- | --- | --- | --- | --- | --- | --- | --- | --- | --- |
|  |  | **Feb** | **May** | **Sep** | **Feb** | **May** | **Sep** | **Feb** | **May** | **Sep** | **Feb** | **May** | **Sep** | **Feb** | **May** | **Sep** | **Feb** | **May** | **Sep** | **Feb** | **May** | **Sep** |  |
| River | BLK | 7.9 | 7.6 | 6.7 | 18.2 | 23.63 | 21.4 | 957.93 | 663.37 | 548.55 | 8.41 | 9.54 | 4.08 | 718.0 | 536.7 | 382.5 | 10.95 | 9.00 | 5.4 | 4.72 | 5.63 | 2.82 | 165.83 |
|  | DES | 6.5 | 6.6 | 6.3 | 15.9 | 23.01 | 19.1 | 929.07 | 828.37 | 187.13 | 3.31 | 1.45 | 3.23 | 730.0 | 601.0 | 137.0 | 5.00 | 6.5 | 4.5 | 7.30 | 10.47 | 7.28 | 157.64 |
|  | DRE | 7.3 | 6.7 | 6.7 | 21.9 | 19.56 | 21.1 | 1459.57 | 574.57 | 479.13 | 10.33 | 3.64 | 5.36 | 1008.0 | 394.0 | 336.3 | 18.50 | 8.9 | 6 | 17.49 | 12.63 | 13.50 | 152.63 |
|  | EFT | 6.8 | 6.5 | 6.6 | 20.5 | 22.31 | 22.2 | 1801.83 | 1210.97 | 915.73 | 0.48 | 1.73 | 0.79 | 1280.0 | 809.0 | 628.0 | 138.50 | 75 | 55.5 | 33.72 | 35.22 | 24.18 | 156.81 |
|  | CRZ | 7.1 | 6.5 | 6.7 | 18.4 | 23.56 | 20.5 | 1249.53 | 382.83 | 429.93 | 4.33 | 4.79 | 5.87 | 928.0 | 259.3 | 305.0 | 14.40 | 5.5 | 5.7 | 13.41 | 16.12 | 9.24 | 135.49 |
|  | TRN | 6.9 | 6.6 | 6.5 | 19.0 | 22.85 | 20.2 | 1327.90 | 533.97 | 543.30 | 6.57 | 3.39 | 5.35 | 974.0 | 368.7 | 388.7 | 36.40 | 24 | 8.2 | 20.43 | 15.28 | 19.60 | 151.88 |
|  | EZP | 7.2 | 7.0 | 6.8 | 18.6 | 21.94 | 20.3 | 1407.80 | 839.27 | 570.93 | 4.32 | 4.07 | 8.86 | 1047.0 | 594.0 | 407.3 | 12.10 | 9.8 | 5.2 | 21.00 | 11.85 | 12.90 | 171.87 |
|  | PSL | 6.8 | 7.2 | 6.7 | 19.4 | 25.71 | 23.0 | 456.13 | 440.20 | 388.00 | 6.37 | 4.92 | 6.03 | 331.0 | 282.0 | 195.5 | 3.50 | 1.6 | 3.9 | 0.17 | 0.71 | 1.31 | 38.22 |
| Lake | BAZ | 7.4 | 7.3 | 7.7 | 20.8 | 25.41 | 23.2 | 465.03 | 435.70 | 387.50 | 9.08 | 5.95 | 9.70 | 329.0 | 281.0 | 261.0 | 1.40 | 1.5 | 2.6 | 2.30 | 0.56 | 1.07 | 38.22 |
|  | CHJ | 7.8 | 8.3 | 7.0 | 21.0 | 25.06 | 23.2 | 866.67 | 646.67 | 498.75 | 10.13 | 9.84 | 5.13 | 713.0 | 584.3 | 336.0 | 2.50 | 5.65 | 4.3 | 3.56 | 4.72 | 1.96 | 165.83 |
|  | CPT | 6.8 | 7.3 | 6.8 | 19.4 | 25.16 | 23.3 | 613.57 | 648.00 | 404.40 | 8.95 | 9.83 | 4.95 | 447.0 | 420.0 | 271.0 | 2.65 | 1.7 | 1.55 | 5.23 | 1.545 | 1.23 | 31.22 |
|  | ECT | 7.2 | 7.6 | 7.5 | 20.1 | 20.72 | 24.9 | 395.20 | 422.63 | 382.00 | 5.71 | 4.07 | 8.86 | 272.0 | 284.0 | 248.7 | 3.80 | 1.9 | 1.9 | 0.73 | 0.615 | 1.67 | 36.78 |
|  | ENS | 7.5 | 7.4 | 7.1 | 20.3 | 25.66 | 24.0 | 270.13 | 295.6 | 246.80 | 9.52 | 6.70 | 6.81 | 193.0 | 272.0 | 163.5 | 1.60 | 2.1 | 2.4 | 0.29 | 0.315 | 0.26 | 0 |
|  | JZN | 7.1 | 7.6 | 7.5 | 18.9 | 24.78 | 22.3 | 389.67 | 415.5 | 353.43 | 9.61 | 7.03 | 6.49 | 267.0 | 281.0 | 242.3 | 1.50 | 1.7 | 1.2 | 0.33 | 1.265 | 0.19 | 143.10 |
|  | POJ | 7.4 | 7.4 | 7.3 | 20.5 | 23.92 | 23.1 | 203.13 | 218.63 | 184.70 | 9.56 | 7.30 | 7.14 | 145.0 | 145.0 | 124.7 | 1.20 | 1.7 | 1.2 | 0.56 | 0.59 | 0.24 | 0 |
|  | SNJ | 7.1 | 7.1 | 7.2 | 20.6 | 25.23 | 24.9 | 365.60 | 369.07 | 273.77 | 9.71 | 7.03 | 6.96 | 259.0 | 239.0 | 178.0 | 2.40 | 2.3 | 1.9 | 1.41 | 1.19 | 1.62 | 73.53 |
|  | SNL | 6.8 | 7.5 | 7.7 | 20.0 | 25.12 | 24.2 | 671.40 | 652.93 | 405.40 | 1.93 | 9.08 | 9.68 | 483.0 | 423.3 | 267.7 | 4.55 | 2.65 | 3.1 | 2.43 | 1.60 | 1.25 | 64.84 |
|  | TZC | 7.4 | 7.4 | 7.4 | 19.1 | 23.62 | 23.1 | 242.27 | 266.97 | 227.17 | 10.14 | 7.38 | 7.45 | 179.0 | 178.0 | 153.0 | 1.60 | 1.9 | 1.5 | 0.54 | 0.625 | 0.67 | 2.88 |

**Supplementary material B.** Morphometric characteristics of the dominant cyanobacteria identified in this study. The figures mentioned within the descriptions can be consulted in the main manuscript.

### *Microcystis* sp. (Fig. 5-a). The specimens form rounded to irregular colonies, without a mucilaginous envelope, composed of spherical cells (diameter = 1.7-5.57 µm) containing gas vesicles. Identifying species within this genus based solely on morphology is highly challenging due to extensive colony variability and high phenotypic plasticity; therefore, the isolate was not assigned a specific epithet.

*Limnothrix* sp*.* (Fig. 5-b)*:* Morphologically, the specimens consist of individual filaments that generally lack a sheath. The vegetative cells range from isodiametric to longer than they are wide (length= 2.9-7.8 µm; width= 1.5-3.7 µm) and are un-constricted at the cross-walls. Typically, the apical cells are cylindrical and lack a calyptra.

*Microcoleus* sp. (Fig 5-c): Morphologically, the isolates form uniseriate filaments with cells usually shorter (length= 3.14-5.88 µm; width= 3.83-6.99 µm) and occasionally isodiametric, enclosed within a mucilaginous tubular sheath that may be lost under culture conditions. Genetically, the sequences obtained from cultures CRZ I, CRZ II, and CRZ III clustered with *Microcoleus* sp., forming a clade supported by a bootstrap value of 77% (Fig. 5). This lineage can be distinctly identified by a molecular autoapomorphy—specifically, an 11-bp insertion in the 16S rRNA gene (Boyer et al., 2002; García-Pichel et al., 2001)—which was confirmed to be present in the analyzed sequences from these cultures.

*Oscillatoria* sp. (Fig. 5-d): The filaments occur solitarily and are straight to slightly curved, exhibiting neither constrictions nor a discernible sheath. The cells are characteristically shorter (1.08 – 1.82 µm) than they are wide (2.9-3.3 µm) and are devoid of gas vesicles. The apical cells are rounded and lack a calyptra. Due to the absence of distinct diagnostic features, multiple taxa conform to this morphological profile.

### *Planktothrix agardhii* (Fig. 5-e): Solitary, straight or slightly curved filaments, cell shorter in width (1.6-3.9 µm) than length (3.1-5.6 µm) with gas vesicles distributed throughout the cell and without a sheath, akinetes, and heterocysts. Molecular analysis of strains identified as *P. agardhii* (B-22, B2-22, C-22, C14-22 and D16-22) are described in Carmona et al., (2024). Morphological genus identification was confirmed by a Maximun likelihood analysis, meanwhile, species differentiation was supported by a dissimilarity analysis of the 16S-23S ITS rRNA.

*Raphidiopsis* sp. (Fig. 5-f): The filaments appear solitary and slightly curved, devoid of a sheath or mucilaginous envelope. The cylindrical vegetative cells are noticeably longer than wide (length = 7.05-11.27 µm; width = 2.39-3.34 µm), while the apical cells are distinctly pointed. Granular cellular inclusions are present, and no heterocytes were observed in the examined specimens.

### *Persinema komarekii* (Fig. 5-g): Morphologically, the isolates present as solitary and straight trichomes. The vegetative cells are cylindrical and elongated (length = 1.91-3.79 µm; width = 1.20-2.37 µm), showing constrictions at the cross-walls and lacking aerotopes; the apical cells are rounded to flattened and do not possess a calyptra. In the phylogenetic analysis (Fig. 6), sequences from the DRN cultures (42.2 and 42.3) clustered within the *Persinema* clade (*Persinema* sp. and *P. komarekii*) with 92% bootstrap support (Fig. 6). However, given the discrepancies with the known ecological characteristics of this genus, further analyses are required to validate its taxonomic identity.

*Anabaenopsis elenkinii* (Fig. 5-h): The trichomes appear solitary or entangled in small clusters; they are typically coiled or spiraled, rarely straight, and lack a distinct sheath. The cylindrical vegetative cells are generally longer (3.6-7.5 µm) than wide (4.73-6.99 µm) and contain facultative gas vesicles. While heterocytes are present, akinetes were not observed in the samples. A diagnostic characteristic of this genus is the development of apical heterocytes, which emerge in pairs subsequent to the asymmetric division of two vegetative cells within the trichome, eventually separating upon trichome fragmentation. Morphologically, this species exhibits a close affinity to *A. arnoldii* (Komárek, 2005)

In the phylogenetic analysis, sequence from culture of CHJ (A1) clustered with *Anabaenopsis elenkinii* (AM773306.1), with 60% bootstrap support. In turn, this clade grouped with a clade that contained a cluster of sequences of this species, supported by a bootstrap value of 89% (Fig. 7).

*Dolichospermum* sp. (Fig. 5-i): Morphologically, the trichomes exhibit a metameric structure with respect to the heterocytes and lack a firm sheath. The cells are isopolar, and rarely wider (length = 3.11-6.03 µm; width =4.24-5.99 µm), containing gas vesicles grouped into aerotopes that are dispersed throughout the cytoplasm. Additionally, the filaments possess intercalary heterocytes, with akinetes developing adjacent to them. This genus displays a strong morphological resemblance to *Anabaenopsis*; however, the latter is distinguished by the presence of apical heterocytes.
